# Impact of education and training on type of care provided by community-based breastfeeding counselors: a cross-sectional study

**DOI:** 10.1186/1746-4358-6-12

**Published:** 2011-08-26

**Authors:** Elizabeth M Sullivan, Whitney E Bignell, Anne Andrianos, Alex K Anderson

**Affiliations:** 1 Department of Foods and Nutrition, University of Georgia, 280 Dawson Hall, Athens, GA 30602, USA; 2 International Board of Lactation Consultant Examiners, 6402 Arlington Boulevard, Suite 350, Falls Church, VA 22042, USA

## Abstract

**Background:**

Studies using community-based breastfeeding counselors (CBBCs) have repeatedly shown positive impact on breastfeeding initiation, exclusivity and duration, particularly among low-income mothers. To date, there has not been a comprehensive study to determine the impact of CBBC attributes such as educational background and training, on the type of care that CBBCs provide.

**Methods:**

This was a cross-sectional study of a convenience sample of CBBCs to ascertain the influence of counselor education and type of training on type of support and proficiency of CBBCs in communities across the United States. Invitations to participate in this online survey of CBBCs were e-mailed to program coordinators of the Special Supplemental Nutrition Program for Women, Infants, and Children (WIC), La Leche League, and other community-based health organizations, who in turn invited and encouraged their CBBCs to participate. Descriptive analysis was used to describe participants (N = 847), while bivariate analysis using χ^2 ^test was used to examine the differences between CBBC education, training received and breastfeeding support skills used. Multivariate logistic regression was used to assess the independent determinants of specific breastfeeding support skills.

**Results:**

The major findings from the research indicate that overall, educational attainment of CBBCs is not a significant predictor for the curriculum used in their training and type of support skills used during counseling sessions, but initial training duration was positively associated with the use of many breastfeeding support skills. Another major influence of counselor support to clients is the type of continuing education they receive after their initial training, with higher likelihood of use of desirable support skills associated with counselors continuing their breastfeeding education at conferences or trainings away from their job sites.

**Conclusions:**

Our results show that different programs use different training curricula to train their CBBCs varying in duration and content. Counselor education is not a significant predictor of the type of training they receive. Continuing breastfeeding education is a significant determinant of type of counseling techniques used with clients. Further research is therefore needed to critically examine the content of the various training curricula of CBBC programs. This may show a need for a standardized training curriculum for all CBBC programs worldwide to make CBBCs more proficient and efficient, ensuring successful and optimum breastfeeding experiences for mothers and their newborns.

## Background

Due to the superiority of human milk over infant formula in the early postpartum period, both the American Academy of Pediatrics (AAP) and the American Dietetic Association (ADA) recommend breastfeeding duration of at least twelve months and exclusive breastfeeding for the first six months after delivery [1-3]. The World Health Organization (WHO) also recommends at least 24 months of breastfeeding with appropriate complementation and 6 months of exclusive breastfeeding as part of the Global Strategy for Infant and Young Child Feeding. In 2009, the Centers for Disease Control and Prevention (CDC) reported that although some specific states, particularly in the west and northeast of the United States, have met or exceeded the breastfeeding goals set forth in *Healthy People 2010*, the nation as a whole fell short of the targets for breastfeeding duration and exclusivity [4,5]. Even though some of the objectives were not met for the *Healthy People 2010 *goals, new and more challenging breastfeeding goals are set forth in *Healthy People 2020*, with the aim to increase breastfeeding rates to the targets of: 82% of mothers to breastfeed their newborns in the early postpartum period, 61% and 34% continue breastfeeding at six months and at one year, respectively, as recommended by the AAP [6]. The fact that not all previous breastfeeding objectives were met despite the enormous effort put into breastfeeding promotion and support in the last decade suggests that pregnant and new mothers may not be getting adequate information about the importance of breastfeeding nor the appropriate support needed to be successful with breastfeeding. While International Board Certified Lactation Consultants (IBCLCs) are extremely valuable resources as knowledgeable breastfeeding supporters, the United States can only boast of approximately 11,000 who are limited to certified Baby Friendly Hospitals and birthing facilities and just a few non-Baby Friendly Hospitals [7,8]. This means they may not be able to reach all populations, particularly those most in need (low-income, immigrants/minorities and residing in deprived communities). In the National Immunization Survey 2004-2008, the CDC found that disparities exist among racial/ethnic groups, with the African-American population having one of the lowest rates of breastfeeding initiation and duration [9]. This is where another form of support may prove vital: community-based breastfeeding counselors (CBBCs).

Community-based Counselors (CBCs) go by many names: peer educators, peer counselors, lay support, community outreach workers, and indigenous health care advisors, among others [10]. The concept of CBC was developed as a means of reaching out to educate, support, advise, and counsel individuals in need of help who otherwise do not have access to the mainstream healthcare services using lay persons [11]. Whether that help relates to depression in seniors, diabetes management, surviving with HIV/AIDS, improving nutrition, recovering from alcohol abuse, coping with breast cancer, or any other health concern, is determined by the program that implements it [12-18]. Over the years, studies have shown time and again that CBC is an exceptionally effective means of implementing positive behavioral and lifestyle changes [10-18].

CBCs have been used to promote breastfeeding by agencies such as the Expanded Food and Nutrition Education Program (EFNEP); the Special Supplemental Nutrition Program for Women, Infants, and Children (WIC); and the Breastfeeding Heritage and Pride Program with great success [19,20]. These community-based breastfeeding counselors have been used extensively and effectively to promote breastfeeding in selected communities, especially low-income minority communities who otherwise do not breastfeed. In 2005, Anderson et al published results from a randomized, controlled trial showing that CBBC positively influences breastfeeding outcomes among low-income Latina and other minority women [21]. Chapman et al performed a systematic review of randomized controlled trials from around the world that evaluated the impact of CBBC on breastfeeding initiation, rates, exclusivity, and maternal and child health outcomes [22]. In their review, the researchers found that interventions that included antenatal and multiple perinatal CBBC contact yielded the most success in increasing breastfeeding initiation rates as compared to controls. Chapman et al also discovered that interventions that improved breastfeeding duration and exclusivity significantly were those that included ongoing, in-person (face-to-face) CBBC support [22]. What makes a peer so successful at giving support are her previous experiences that she can share and relate to her client, which in turn helps the client to open up about themselves and build rapport. CBBCs have repeatedly produced positive outcomes regarding breastfeeding, particularly with regards to increasing the rate of breastfeeding initiation and exclusivity, as well as extending breastfeeding duration [19,21,23,24]. In many cases, a CBBC is recruited from the target population/community, which allows them to identify and reach people who may otherwise be isolated, underserved, or potentially unaware of the services and information available to them [15].

Sufficient training is imperative to a successful program with positive health outcomes, and in the case of breastfeeding, increasing the proportion of mothers initiating, as well as extending, the duration of breastfeeding [20,24-26]. According to Rossman (p. 633), CBBC training programs are designed in a way to "orient the counselor to program objectives, to promote the attainment of skills that enable the effective use of their breastfeeding knowledge, and to promote understanding of the needs of the target population," which should include the actual physiology of breastfeeding, hands-on support techniques, positive counseling skills, and cultural understanding [27]. As mentioned previously, breastfeeding is an area in which CBBCs have proven to be very effective; however, there is a lack of consistency in training curriculum from program to program. There are differences in knowledge and proficiency between a skilled professional and a peer educator, which can be exacerbated if inadequate training is provided [17]. When counseling does not provide capable support in breastfeeding, there is a higher risk of failure in implementing good breastfeeding practices by mothers [28]. The World Health Organization (WHO) set the gold-standard for CBBC training programs at 40 hours duration, although they recommend a minimum of 18 hours with an additional 3 hours of hands-on practice [29]. This suggests the importance of hands-on practice in the training of CBBCs. Although both the California Department of Health's WIC-based breastfeeding peer counseling training program and the one offered by La Leche League International are approximately the same in duration at around 20 hours, content can vary with location, program, state, or even country [30,31].

Rossman and Chapman et al in their systematic reviews of numerous CBBC studies/programs, demonstrated the variability among the training protocols used by different programs [22,27]. It was determined in both of these reviews that the duration of training varies from 9 hours to 56 hours, depending upon whether the program was based on the World Health Organization model or was developed strictly for research purposes. Bronner et al conducted a national survey of WIC, one of the largest employers of breastfeeding counselors, regarding their role at the state and local levels within the organization [32]. Although the findings of Bronner et al support other studies that have established the effectiveness of CBBCs in breastfeeding promotion, they found that there was a lack of consistency in the recruitment, training, and counseling practices of counselors across WIC agencies in the United States [32]. These findings therefore suggest a need for standardization of training and counseling protocols for CBBC programs across the United States and the world in general.

Advantages to standardized training programs such as those offered by La Leche League or WHO/UNICEF would include ensuring consistent, accurate, and up-to-date information regarding benefits and techniques of breastfeeding passed along from CBBCs to new mothers, as well as making certain that the counseling techniques used by the CBBCs are appropriate and correct [33]. Through training, CBBCs need to acquire science-based practical knowledge about breastfeeding as well as be able to consider their own experiences and beliefs. These will contribute to the development of their interpersonal skills that they will use when counseling new mothers [34].

While extensive research has been done to support the importance of CBBCs, to date there has not been a comprehensive nationwide study to assess the impact of CBBC attributes, such as CBBC-educational background and CBBC-training, on the type of care that CBBCs provide. The objectives of this study are to examine the influence of a) CBBC formal educational background on the type of training they receive to become counselors and b) CBBC formal educational background and training on the proficiency and type of breastfeeding support they provide their clients.

## Methods

### Study design and participants

This was a cross-sectional study hosted online through SurveyMonkey^® ^of a convenience sample of CBBCs to examine the association between formal educational background and type of training on type of support and proficiency of CBBCs in communities across the United States. In all, we received 1,030 responses, of which 183 were excluded, leaving 847 participants included in our final analysis. Participants were para-professionals/lay women 18 years of age or older trained to provide breastfeeding education/counseling and support to pregnant and breastfeeding mothers in communities across the United States. Respondents were excluded from the study if they indicated they possessed any sort of breastfeeding certification or professional breastfeeding credential, such as Certified Lactation Counselor (CLC) or International Board Certified Lactation Consultant (IBCLC). This was to ensure that the participants were indeed lay people. By definition, a para-professional is one trained in a particular field to assist a professional, but is not themselves certified or licensed in that profession [35]. Respondent data was also excluded from the final analysis if they were missing any demographic information.

### Questionnaire development and testing

A structured questionnaire consisting of 35 questions was developed to collect information about employment status (paid or volunteer, benefits, pay rate, agency/organization), training information (course duration, continuing education, hands-on practice [observe mothers breastfeed, position and correct poor latch of baby at the breast under some degree of supervision]), counseling skills (hands-on support, demonstrations for the clients, active listening, referrals to IBCLC when needed), counseling settings (face-to-face, by phone, internet, mail, group sessions, in the hospital, home visits), topics discussed during counseling (breastfeeding problems and solutions, pumping and storage of breast milk, solutions for working mothers, maternal nutrition, child health, medications during breastfeeding), management and care of cases, experience, education, demographics, and race/ethnicity (counselors and clients) of CBBCs. The principal investigators, familiar with breastfeeding education and CBBC training program curricula, in consultation with a psychometrician, reviewed and developed the survey tool. As this survey was a first of its kind, an extensive review of the literature and many interviews with CBBC program managers and trainers were conducted. The draft survey was subsequently reviewed by several experts, representing various disciplines including human lactation, education, dietetics and nursing. The draft questionnaire was pilot tested with practicing community-based breastfeeding counselors (N = 11) for content validity. These same community-based breastfeeding counselors also participated in an in-depth interview to ascertain the literacy level and appropriateness of tasks (counseling and management). After further review of the draft questionnaire, the investigators came to a consensus and finalized the survey for distribution to participants.

### Survey distribution

Invitations with a link to the survey hosted by SurveyMonkey^® ^were e-mailed to program coordinators of WIC, La Leche League, Cooperative Extension, Early Head Start, Healthy Start, Gift Project, Connect One Chicago, Breastfeeding Heritage and Pride of the Hispanic Health Council (Hartford, CT), Doulas of North America, Nursing Mothers Council, and Nursing Mothers Network, which all provide community-based breastfeeding counseling services. These programs, using lay women and para-professionals who receive some training in breastfeeding education/support and are paid or are volunteers, were identified through initial literature reviews and personal conversations with experts in the field of breastfeeding during our survey development. The program coordinators of the above mentioned programs across the United States, then invited and encouraged their CBBCs to participate. Besides the initial email invitation to program coordinators, another email reminder was sent to coordinators to encourage their CBBCs to complete the survey online through SurveyMonkey^®^. Email invitations were sent to program leaders in the 50 states, the District of Columbia and Puerto Rico. Consent of participants was obtained through clicking "Yes" to the first question, "I agree to participate in this survey," after reading a consent script describing the survey and the responsibility of the respondent. Completed surveys were captured by SurveyMonkey^® ^and downloaded by the researchers for analysis. All methods and procedures were approved by the University of Georgia Institutional Review Board.

### Analysis

All data was downloaded from SurveyMonkey and imported into SPSS for Windows (version 17.0) for coding and all analysis. Descriptive statistics were used to describe our participants, with results presented as percentages and Figures. Bivariate analysis using χ^2 ^test was used to examine differences between breastfeeding support skills, CBBC education level and type of training received. Due to the categorical nature of the data, logistic regression was used to assess determinants of specific breastfeeding support skills. Univariate logistic regression was performed on each of the dependent variables with each of the independent variables. The independent variables that were found to be significantly associated with each of the dependent variables were subsequently incorporated into the multivariate logistic regression models and reported in the paper. Independent variables primarily consisted of demographic information, initial breastfeeding training curriculum (duration), and continuing breastfeeding education, while the dependent variables chosen were those that we determined to best represent different aspects of breastfeeding counseling: hands-on support skills, face-to-face counseling, client-centered counseling skills, and referrals to healthcare professionals, in addition to hands-on practice during training. A p-value of ≤ .05 was used as the criterion for statistical significance.

## Results

### Participant characteristics

Table 1 presents the characteristics of the survey participants. Over a third (35.2%) of the participants were between 30-39 years while 5.4% reported to be 60 years or older. The majority (74.9%) of counselors self-identified their race/ethnicities as white/Caucasian with only 7.6% as black/African American. In contrast, the reported race/ethnicities of the clientele served by the counselors were as follows: 45.3% white/Caucasian, 18.0% Latino/Hispanic, 16.6% black/African American, and the remaining 20.0% belonging to other racial/ethnic groups. Almost two-thirds of the participants reported to be paid counselors, while 36.2% said to be volunteer/unpaid counselors. The majority of the participants reported to be affiliated with La Leche League (30.6%) and WIC (26.4%), while 90.3% had post-high school education (Table 1). Over 70.0% of the participating counselors reported to have 12 months or more personal experience breastfeeding a child or children, while 8.4% did not have any personal breastfeeding experience (Table 1). Regarding initial training to become a CBBC, the majority of counselors (58.8%) reported that their training consisted of several courses/classes over time, while 8.1% received a training lasting less than 10 hours in duration. Sixty-one percent of respondents (61.0%) stated that their training did include hands-on practice or observing mothers and babies feeding up close (Table 1). Over half (52.1%) of the participants reported to have at least five years experience as either a paid or volunteer breastfeeding counselor assisting pregnant and nursing mothers, while 9.9% have been counselors for less than one year. A majority of the counselors reported to have an IBCLC on-site (30.7%) or within a five mile radius (30.8%) for referral of clientele in situations where they encounter a client with significant lactation problems beyond their skill level and needing an expert counsel and support. Over a third (38.1%) of counselors declared that they continue their breastfeeding education by going to conferences and training away from their jobs, while 4.7% have had no continuing education since the initial breastfeeding training (Table 1).

**Table 1:** Characteristics of breastfeeding counselors across the United States (N = 847)

	n	%
Age		
Under 30 years	139	16.4
30-39 years	298	35.2
40-49 years	202	23.8
50-59 years	162	19.1
Over sixty years	46	5.4

College education		
Did not attend college	82	9.7
Attended but did not complete college	265	31.3
Completed college	500	59.0

Counselor race/ethnicity		
Asian American	5	0.6
Black/African American	64	7.6
Latino/Hispanic American	92	10.9
Native American	7	0.8
Multiracial American	17	2.0
White/Caucasian	634	74.9
Other	28	3.3

Race/ethnicity of clientele		
Asian American	75	8.9
Black/African American	230	27.2
Latino/Hispanic American	250	29.5
Native American	31	3.7
Multiracial American	143	16.9
White/Caucasian	628	74.1
Other	27	3.2

Position of breastfeeding work		
Paid full-time	236	27.9
Paid part-time	304	35.9
Volunteer	307	36.2

Training duration		
< 10 hour course	69	8.1
10-20 hour course	106	12.5
>20 hour course	174	20.5
Several classes over time	498	58.8

Training included hands-on practice?		
Yes	517	61.0
No	330	39.0

Continuing education		
One conference a year	65	7.7
Training at job	339	40.0
Conferences and training away from job	323	38.1
Study on own time	80	9.5
No additional training or education	40	4.7

Place of work or volunteer		
Community/Public Health Clinic	128	15.1
Healthy Start	50	5.9
Hospital	28	3.3
WIC	224	26.4
Breastfeeding support group (like LLL)	259	30.6
Doula	11	1.3
Other*	147	17.4

Work setting		
Rural: < 10,000 people	124	14.6
Small city: 10-100,000 people	240	28.3
Medium city: 100-500,000 people	191	22.6
Urban large city: >500,000 people	179	21.1
Suburban near a medium or large city	113	13.3

How close is the nearest IBCLC for referral?		
IBCLC at site	260	30.7
< 5 miles	261	30.8
6-25 miles	244	28.8
26-50 miles	47	5.5
>50 miles	35	4.1

Past personal breastfeeding experience		
Did not breastfeed	71	8.4
1-4 months	37	4.4
5-6 months	25	3.0
6-12 months	87	10.3
12-24 months	445	52.5
>24 months	182	21.5

Duration helping breastfeeding mothers		
< 1 year	84	9.9
1-4 years	322	38.0
5-9 years	191	22.6
10-15 years	104	12.3
>15 years	146	17.2

*Mother-Baby Group, Mothering Support Group, Holistic Moms, New Mothers Internet Group, Maternity Retail Store, Connect One Chicago, Gift Project, etc.

### Educational attainment of CBBCs and breastfeeding support skills

**Figure 1:**
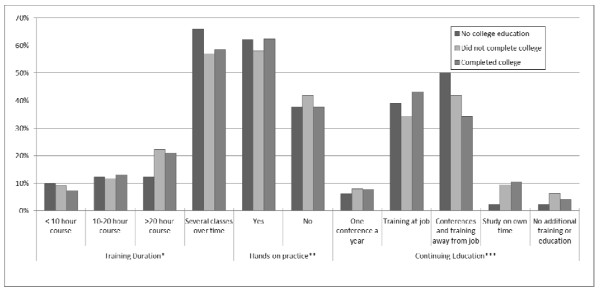
Association of educational attainment with type of initial training, hands-on practice and continuing education *p = 0.522, **p = 0.499, ***p = 0.025

We examined the associations between participants' educational attainment and a number of variables, such as the duration and type of training received as well as the use of specific counseling characteristics (Figure 1 and Table 2). There was no significant association between the use and frequency of hands-on support during counseling sessions, or face-to-face counseling at place of work/organization and education of participants. Use of client-centered counseling skills with pregnant and breastfeeding mothers was found to be significantly associated with educational background of participants (p = 0.002) with about 90% of those with no college education and some college education using these skills unlike their counterparts with a college degree. There was a significant inverse association between educational attainment and whether counselors reported reading instructions and writing out forms for clients who have trouble doing so (p < 0.001), identifying social service needs (p = 0.001) and referring mothers to social service agencies (p = 0.003), as well as referring mothers to community resources such as WIC, food banks, or job training programs (p = 0.001) (Table 2). Educational attainment of the counselor was not a significant independent predictor for any of the counseling skills employed by participants in this study.

### Initial breastfeeding training of CBBCs and support skills

**Figure 2:**
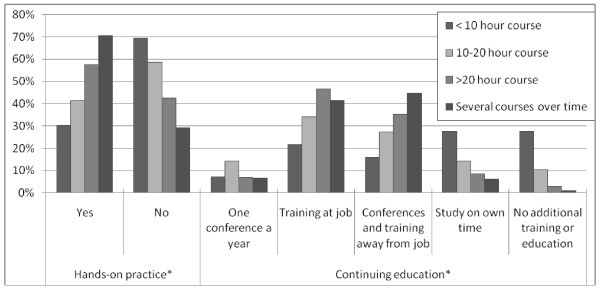
Association of initial training duration with hands-on practice and continuing education *p < 0.001

We observed a significant association between type of training received and hands-on practice during training (Figure 2 and Table 3) with a majority (70.7%) of those whose training involved several courses/classes over time also receiving hands-on practice during training (p < 0.001 ). All of the variables related to hands-on support given to mothers during counseling were found to be significantly associated with training duration (p < 0.001), with longer training duration being positively associated with more frequent use of each skill: help mother position baby at the breast, observe mother breastfeed her baby, and correct baby's poor latch to the breast. There was also a significant positive association of longer training duration with frequency of counseling mothers face-to-face at the counselor's place of work/organization (p < 0.001), as well as referral of mothers to a healthcare professional including IBCLC for additional support (p = 0.007) (Table 3).

**Table 2:** Association of counseling characteristics and educational attainment (N = 847)

	No college education n (%)	Did not complete college n (%)	Completed college n (%)	p-value
***Hands-on support for mothers during a counseling session:***				
Help mother to position baby at the breast?				0.146
Never	15 (18.3)	27 (10.2)	64 (12.8)	
Did do	67 (81.7)	238 (89.8)	436 (87.2)	

Observe mother breastfeeding her baby?				0.759
Never	6 (7.3)	23 (8.7)	36 (7.2)	
Did do	76 (92.7)	242 (91.3)	464 (92.8)	

Correct a baby's poor latch at the breast?				0.358
Never	24 (29.3)	58 (21.9)	125 (25.0)	
Did do	58 (70.8)	207 (78.1)	375 (75.0)	

***Face-to-face sessions with mothers:***				
Counsel mothers face-to-face at your place of work?				0.052
Never	5 (6.1)	29 (10.9)	74 (14.8)	
Did counsel	77 (93.9)	236 (89.1)	426 (85.2)	

***Counseling skills used:***				
Use client-centered counseling skills with pregnant and breastfeeding women?				0.002
Never	10 (12.2)	34 (12.8)	111 (22.2)	
Did use	72 (87.8)	231 (87.2)	389 (77.8)	

***Referral to a healthcare professional (includes IBCLC):***				
Refer mother to health care professionals?				0.531
Never	3 (3.7)	5 (1.9)	9 (1.8)	
Did refer	79 (96.3)	260 (98.1)	491 (98.2)	

***Other services or skills:***				
Plan and teach a back to work/school breastfeeding classes?				< 0.001
Never	53 (64.6)	151 (57.0)	361 (72.2)	
Did plan and teach	29 (35.4)	114 (43.0)	139 (27.8)	

Read instructions or directions for clients who are unable to read well?				< 0.001
Never	20 (24.4)	64 (24.2)	224 (44.8)	
Did read	62 (75.6)	201 (75.8)	276 (55.2)	

Write out forms for clients who cannot do so?				< 0.001
Never	30 (36.6)	117 (44.2)	318 (63.6)	
Did write	52 (63.4)	148 (55.8)	182 (36.4)	

Identify social service needs (such as housing or food)?				0.001
Never	18 (22.0)	66 (24.9)	180 (36.0)	
Did identify	64 (78.0)	199 (75.1)	320 (64.0)	

Encourage or make referrals to social service agencies?				0.003
Never	11 (13.4)	43 (16.2)	126 (25.2)	
Did encourage	71 (86.6)	222 (83.8)	374 (74.8)	

Refer mothers to community resources (such as WIC or food bank)?				0.001
Never	6 (7.3)	24 (9.1)	90 (18.0)	
Did refer	76 (92.7)	241 (90.9)	410 (82.0)	

In our multivariate logistic regression analysis, duration of the training curricula used by the CBBC program and proximity of IBCLC to the place of work/organization were significant independent predictors for counselors having hands-on practice during their training to become CBBCs after adjusting for other covariates. Compared to counselors whose training curricula was made up of several classes over time, those whose training involved any of the other training curricula were less likely to have had any hands-on practice during their initial breastfeeding training (Table 4) in the order, less than 10 hours (OR = 0.22, 95% CI = 0.12, 0.39), 10-20 hour course (OR = 0.29, 95% CI = 0.19, 0.46), then more than 20 hours (OR = 0.55, 95% CI = 0.38, 0.80). Counselors who had access to IBCLC at their work site were more likely to have had hands-on practice during their training, with this likelihood decreasing as the distance to the nearest IBCLC increased with respect to the counselors place of work/organization (Table 4).

**Table 3:** Association of counseling characteristics and duration of training (N = 847)

	A training or course <10 hours n (%)	10-20 hour training or course n (%)	A training or course >20 hours n (%)	Several trainings or courses over time n (%)	p-value
*Hands-on support for mothers during a counseling session:*					
Help mother position baby at the breast?					< 0.001
Never	15 (21.7)	27 (25.5)	25 (14.4)	39 (7.8)	
Did do	54 (78.3)	79 (74.5)	149 (85.6)	459 (92.2)	
Observe mother breastfeed her baby?					< 0.001
Never	12 (17.4)	22 (20.8)	13 (7.5)	18 (3.6)	
Did do	57 (82.6)	84 (79.2)	161 (92.5)	480 (96.4)	

Correct baby's poor latch at the breast?					< 0.001
Never	26 (37.7)	41 (38.7)	39 (22.4)	101 (20.3)	
Did do	43 (62.3)	65 (61.3)	135 (77.6)	397 (79.7)	

***Face-to-face sessions with mothers:***					
Counsel mothers face-to-face at your place of work?					< 0.001
Never	17 (24.6)	18 (17.0)	34 (19.5)	39 (7.8)	
Did do	52 (75.4)	88 (83.0)	140 (80.5)	459 (92.2)	

***Counseling skills used:***					
Did you use client-centered counseling skills with pregnant and breastfeeding women?					0.269
Never	18 (26.1)	16 (15.1)	34 (19.5)	87 (17.5)	
Did do	51 (73.9)	90 (84.9)	140 (80.5)	411 (82.5)	

***Referral to a healthcare professional (includes IBCLC):***					
Refer mother to health care professionals?					0.007
Never	5 (7.2)	1 (0.9)	1 (0.6)	10 (2.0)	
Did do	64 (92.8)	105 (99.1)	173 (99.4)	488 (98.0)	

### Continuing breastfeeding education of CBBCs and support skills

For counselors to correct a poor latch by the baby at the breast in our multivariate logistic regression (Table 5), continued breastfeeding education, personal breastfeeding experience and race/ethnicity of counselors were the significant independent predictors in the presence of other covariates. For example, participants who continued their breastfeeding education by attending conferences or trainings away from their job/organization were over six times more likely, as compared to their counterparts who have not had any form of continuing education, to correct a poor latch by the baby at the breast (Table 5). Continuing breastfeeding education was also a significant independent predictor for other forms of hands-on support, such as helping mothers to position baby at the breast and observing mothers breastfeeding their babies (Table 5).

**Table 4:** Factors associated with hands-on practice during training: multivariate logistic regression*

	n	OR	95% CI	p-value
Training duration				
< 10 hour course	69	0.22	0.12, 0.39	< 0.001
10-20 hour course	106	0.29	0.19, 0.46	< 0.001
>20 hour course	174	0.55	0.38, 0.80	0.002
Several classes over time	498	1.00		

How close is the nearest IBCLC for referral?				
IBCLC at site	260	3.38	1.51, 7.53	0.003
< 5 miles	261	2.32	1.05, 5.14	0.038
6-25 miles	244	3.56	1.60, 7.96	0.002
26-50 miles	47	3.16	1.18, 8.43	0.022
>50 miles	35	1.00		

Past personal breastfeeding experience				
Did not breastfeed	71	0.54	0.30, 1.00	0.048
1-4 months	37	0.32	0.14, 0.72	0.006
5-6 months	25	0.52	0.21, 1.31	0.165
6-12 months	87	0.82	0.46, 1.45	0.494
12-24 months	445	0.93	0.63, 1.36	0.692
>24 months	182	1.00		

Counselor race/ethnicity				
Black/African American	64	0.50	0.20, 1.24	0.136
Latino/Hispanic American	92	0.76	0.32, 1.82	0.532
Multiracial American	17	1.20	0.29, 5.00	0.805
White/Caucasian	634	0.49	0.22, 1.06	0.068
Other	40	1.00		

College education				
Did not attend	82	0.91	0.53, 1.55	0.726
Attended but did not complete	265	0.80	0.57, 1.11	0.177
Completed college	500	1.00		

Hosmer and Lemeshow Test of goodness-of-fit: Chi-square = 6.411; p = .601

*Controlled for duration helping breastfeeding mothers and employment status of counselor (paid/volunteer)

**Table 5:** Association between ccontinuing education and use of intensive breastfeeding support during counseling: multivariate logistic regression

		**Observe mothers breastfeed their babies**^**1**^		**Correct poor latch by the baby at the breast**^**2**^		**Use client-centered counseling skills**^**3**^		**Refer mothers to healthcare professional when there is a need**^**4**^	
	**n**	**OR**	**95% CI**	**OR**	**95% CI**	**OR**	**95% CI**	**OR**	**95% CI**

**One conference a year**	65	4.11**	1.15, 14.71	3.85*	1.57, 9.41	4.62*	1.58, 13.51	7.40	0.53, 103.23
**Training at job**	339	3.80*	1.44, 10.04	4.16*	2.01, 8.62	3.06*	1.33, 7.07	13.51**	1.66, 110.12
**Conferences and training away from job**	323	4.30*	1.59, 11.63	6.40*	3.09, 13.28	4.26*	1.81, 10.06	7.58***	0.91, 63.47
**Study on own time**	80	9.51*	2.54, 35.54	4.29*	1.84, 10.04	1.22	0.49, 3.05	6.05	0.67, 54.66
**No additional training or education**	40	1.00		1.00		1.00		1.00	

*p < 0.01, **p < 0.05, ***p < 0.10

^1^Hosmer and Lemeshow Test of goodness-of-fit: Chi-square = 3.129; P = 0.926; controlled for training duration, duration helping breastfeeding mothers, counselor age, education level

^2^Hosmer and Lemeshow Test of goodness-of-fit: Chi-square = 3.981; P = 0.859; controlled for past breastfeeding experience, counselor race/ethnicity, education level

^3^Hosmer and Lemeshow Test of goodness-of-fit: Chi-square = 5.918; P = 0.656; controlled for employment status (paid/volunteer), place of work, education level

^4^Hosmer and Lemeshow Test of goodness-of-fit: Chi-square = 1.399; P = 0.994; controlled for employment status (paid/volunteer), training duration, duration helping breastfeeding mothers, education level

## Discussion

Our results show that race/ethnicities of the CBBCs in breastfeeding support programs across the US is different from the race/ethnicities of the clientele they serve. While the majority (74.9%) of the counselors surveyed identified their race/ethnicities as white/Caucasian, only 45.3% of the clientele they serve were white/Caucasian (Table 1). This is an indication that counselors may generally serve clientele of different racial/ethnic groups other than their own. This finding is a confirmation of earlier results by Bronner et al [32]. In their national WIC survey, Bronner et al reported that 70% of peer counselors identify themselves as Caucasian while only 44% of the clientele pool were Caucasians, which is consistent with our findings.

### Educational attainment of CBBCs and breastfeeding support skills

The majority (90.3%) of the counselors that were surveyed reported having post-high school education (59.0% completed college, 31.3% attended but did not complete college) while 9.7% did not have any college education (only high school education). Typically, community-based counselors are chosen from the target populations they are attempting to serve, and in the case of breastfeeding counseling, programs (such as WIC and EFNEP) are targeted at low-income and minority women who have a higher tendency not to breastfeed and often have no more than high school education [21,36-38]. However, several other agencies (such as La Leche League International, the Massachusetts Breastfeeding Coalition, and the Hispanic Health Council and the Hartford Hospital Breastfeeding Heritage and Pride Program) target women from all walks of life, hence they may recruit counselors with diverse educational backgrounds [38]. In the study by Bronner et al comprising of 663 respondents from 37 states, 59.5% of peer counselors surveyed either attended or completed college, while the median years of education for WIC participants was 12 years, which is slightly different from the findings from the current study [32]. This shows that overall, WIC counselors generally have lower educational levels as compared to the average breastfeeding counselor in the United States. While most CBBC programs require their counselors to have personal breastfeeding experience, others consider passion to help pregnant and new mothers breastfeed successfully as important, which is reflected in the current study with 8.4% of our study participants having no past personal breastfeeding experience.

There were some interesting findings from our research regarding frequency of use of specific support skills with regard to educational attainment of CBBCs that may speak to the ability of counselors to relate to their clientele. Surprisingly, a higher proportion of counselors that completed college reported that they never planned or taught a back-to-work/school breastfeeding class, read instructions or directions for clients who were unable to read well, wrote out forms for clients who could not do so, identified social service needs such as housing, encouraged or made referrals to social service agencies, nor referred mothers to community resources (Table 2). Although providing these services is not a requirement, nor an expectation of the counselors' job description, it is an important way of building rapport with clients. When working with lower-income and minority mothers, such as with WIC or EFNEP clients and those from inner-cities, the inability of counselors to identify with their clients may cause a disconnect in the counselor's ability to identify breastfeeding barriers and needs of the mothers compared to someone who would be more demographically similar to their clients with regard to cultural understanding and understanding the challenges that accompany living below the poverty line or being a minority. It is important to mention that the lack of provision of some of the services listed above may be as a result of the clientele served by CBBCs and not necessarily an inadequacy. As seen from this study, our participants have, on average, higher education backgrounds compared to a typical WIC counselor who is likely to serve clientele who may need such assistance.

To the best of our knowledge, this is the very first national survey of breastfeeding counselors that has examined the association between the educational attainment of counselors and the type of training received, types of breastfeeding support they provide pregnant and nursing mothers. The role of education in the type of training received as well as type and content of support provided by counselors to clients is very important to understand, as different programs struggle with what should be the minimum level of education when it comes to peer counselors. Interestingly, our findings suggest the educational background of the counselor is not as important as the type of initial training and continuing breastfeeding education they receive to be an effective and successful counselor in breastfeeding promotion and support.

### Initial breastfeeding training of CBBCs and support skills

Although a majority (79.3%) of the participants in the current study reported the curriculum used for their initial breastfeeding training was within the minimum duration of 20 hours as recommended by the World Health Organization (WHO), only 61.0% reported their training included hands-on practice, which is an important requirement by the WHO [39]. Most importantly, 70.7% of participants who reported their initial training to consist of several courses/classes over time included hands-on practice, while only 37.1% of those whose training was less than 20 hours in duration had hands-on practice (Table 3). This finding suggests that while the different training curricula for the many CBBC programs may provide similar basic content, more advanced training techniques, such as providing hands-on practice may only occur in longer duration training curricula that occur over several classes, since they would have more time to cover more topics and do this more extensively. Regarding the content of the curricular used to train WIC counselors, Bronner et al reported that the most common topics covered in CBBC training were breastfeeding benefits, common breastfeeding problems, latching on, pumping and storing breastmilk, and counseling techniques [32]. There was no mention in their study of whether WIC included hands-on practice as a part of their training curriculum, although our communication and observation of one of the WIC peer counselor programs in the state of Georgia shows hands-on practice to be part of their training curriculum. This inconsistency may be a reason for the overall low breastfeeding rates and duration among WIC recipients compared to non-WIC recipients even after the introduction of breastfeeding peer counseling services into the WIC program, although this was not assessed in the current study. Providing hands-on practice during the training of counselors as recommended by the WHO is an important way of building the self-confidence of counselors to be able to effectively work with their clients and provide hands-on support during counseling.

The systematic review by Chapman et al took a closer look at the training of the CBBCs around the world [22]. Of the 16 studies examined regarding training, only five reported hands-on practice during the initial breastfeeding training, while the others did not specify at all whether hands-on practice was part of their training curricula. In addition, the scope of training protocol for CBBCs participating in the programs ranged anywhere from 9 hours in duration to over 40 hours. Two studies used the WHO/UNICEF 40-hour model as their basis for training that included hands-on practice, one utilized the WHO 18-hour course, two used La Leche League curricula that included hands-on practice, and seven of the remaining studies included a training duration of 20 hours or more, but with limited details about the actual training protocol [21,22,40-43]. These inconsistencies in the training of CBBCs between programs could explain why efforts to improve breastfeeding rates have been only partially successful in the United States, particularly meeting the *Healthy People 2010 *breastfeeding goals [44,45]. In our univariate logistic regression analysis, we found that counselors whose initial breastfeeding training consisted of twenty hours or less in duration were significantly less likely to have had hands-on practice during their training than those whose training was in the form of several courses/classes over time (data not shown). This was again found to be a significant independent predictor of having hands-on practice during training in the multivariate logistic regression analysis in the presence of other covariates (Table 4). Having hands-on practice during training could be a means of building the CBBCs self-efficacy and self-confidence and being able to perform these tasks with new mothers during counseling sessions when the need arises. We also examined the association between duration of initial CBBC training and use of specific hands-on support techniques during counseling, such as helping mothers to position babies at the breast and correct poor latches by babies at the breast. We observed a significant positive association between training duration and use of these forms of hands-on support by CBBCs while counseling new mothers (Table 3). In a qualitative study by Raisler that investigated the perceptions of mothers participating in Michigan WIC Programs concerning their experiences with CBBCs, she found that technical assistance provided by CBBCs most frequently reported and most valued by the mothers was that of hands-on support in the form of helping with latching and positioning the baby at the breast [33]. Our data shows the significance of comprehensive initial training to ensure the improvement in counselor self-efficacy that can potentially translate to the use of more effective counseling skills such as hands-on support as reported by Rossman [27]. Another important finding from this study is the association between the proximity of IBCLC to job/organization and receiving hands-on practice during the initial breastfeeding training. This finding suggests the importance of CBBCs program structure to involve IBCLCs to ensure the adequacy of the training and the competency of breastfeeding counselors.

### Continuing breastfeeding education of CBBCs and support skills

Continuing breastfeeding education of CBBCs is also an important aspect of increasing counselor efficacy found in this study. This ensures that the CBBCs receive up-to-date, accurate, and relevant information, as well as an opportunity for counselors to improve their own breastfeeding support skills or even learn new ones. In our regression analysis, we found that those who continued their breastfeeding education, particularly in the form of participating in conferences or training away from the job site or studying on their own time, were much more likely than their counterparts who did not continue their breastfeeding education to provide hands-on support in the form of correcting babies' poor latch at the breast during counseling sessions (Table 5). Those who reported continuing their breastfeeding education as part of training at the job site or attending one conference a year were also more likely to report the use of these techniques. Chapman et al, who examined the efficacy of different formats of CBBC counseling interventions with new mothers, reported on higher-intensity interventions (often including numerous pre- and post-natal home or clinic visits) versus lower-intensity interventions (often centered on telephone-based counseling and fewer in-person visits) and their effects on different aspects of improving breastfeeding rates [22]. What the researchers found was that overall, telephone-based counseling methods were among the least effective counseling techniques in improving breastfeeding initiation, duration, and exclusivity [22]. This suggests that face-to-face counseling, whether in mothers' homes, in a clinic, or in CBBC office settings especially if they include hands-on support, are instrumental for reinforcing positive breastfeeding habits in new mothers.

### Strengths and limitations

There are some limitations to this study. First, there is not a way to determine a response rate to this survey, since the number of people to whom the survey was ultimately forwarded to, as well as the number of CBBCs in the United States, is not known. Also, all data were self-reported by the CBBCs with no way to ascertain their validity or otherwise. There was not a "Not applicable" response provided on the survey, so if a question did not apply to the respondent, she could have left the question blank (leading to exclusion from the final data analysis) or potentially responded inappropriately such as "Never." Lastly, there was not a question included in the survey pertaining to the region, state or location of the respondent; hence the data could not be analyzed by state or locality. Also, we were unable to examine our data by the income status of clientele served by our respondents, as some CBBCs served clientele irrespective of their income. Even though WIC breastfeeding counselors are known to serve low-income clients, the clientele pool of the other agencies and organization is difficult to tell, hence our inability to examine the potential association between the support skills reported with the clientele served. This information may have been useful in comparison to known breastfeeding trends, which differ by region of the country and income level.

Besides the limitations mentioned above, this study also has many strengths. We had a very large sample size to work with (847 respondents with complete data), and this gave us a higher statistical power to detect small differences between groups that would not be possible with a smaller sample size. We were also able to distribute the survey nationwide, to all 50 states as well as the District of Columbia and Puerto Rico, and across the CBBC program spectrum by including program coordinators of WIC, La Leche League, Cooperative Extension, Early Head Start, Healthy Start, Gift Project, Connect One Chicago, Doulas of North America, Nursing Mothers Council, and Nursing Mothers Network, therefore allowing generalization of our findings to breastfeeding counselors across the United States.

## Conclusions

This is the very first comprehensive nationwide study to examine the association between CBBC attributes such as CBBC-education and CBBC-training on the type of care and support that CBBCs provide. This research provides preliminary data that allows for the examination of particular CBBC attributes that may have the greatest effect on the type of support they provide to pregnant and breastfeeding mothers. The educational background of counselors is not a determinant of the type of training they receive to become a breastfeeding counselor. Different programs utilize different training curricula for their counselors, with differences in content and duration. We observed a strong association between duration of training, content of training curriculum, and the types of support CBBCs provide their clients. Our findings call for CBBC programs across the United States to consider adopting a training curriculum that is capable of providing counselors the needed competencies, such as providing hands-on support, utilizing client-centered counseling skills, counseling face-to-face, and providing referrals to IBCLC when needed, to make them more efficient in serving their clients. This, therefore, calls for standardization of CBBC training curricula to make them more effective and successful at breastfeeding promotion and support. According to our findings, the standardized training curricula should be close to the WHO recommended training protocol (about 40 hours), with a significant amount of time devoted to hands-on practice and demonstration. Also, there is the need for CBBC programs to encourage and support their counselors to seek continuing breastfeeding education as a means of updating their breastfeeding knowledge and proficiency. Having all of these in place could ensure successful and optimum breastfeeding experiences for mothers and their newborns. Our findings should be interpreted with caution due to the cross-sectional nature of the study, lack of information on the clientele served by the different CBBC programs besides race/ethnicity, scope of data collection and our inability to ascertain the proportion of CBBCs practicing in the United States who responded to the survey.

## Competing interests

The authors declare that they have no competing interests.

## Authors' contributions

EMS participated in data entry, coding, analysis and data interpretation as well as drafting the initial manuscript. WEB participated in data entry and contributed to drafting of the initial manuscript. AA conceived the study and made substantial contributions to its design, coordination, data acquisition and critical review of the manuscript for intellectual content. AKA made substantial contribution to the conception and design of the study, acquisition of data, data analysis and interpretation, manuscript drafting and critical review of the manuscript for intellectual content. All authors read and approved the final manuscript.

## References

[B1] American Academy of Pediatrics (AAP) Work Group on Breastfeeding Breastfeeding and the use of human milk Pediatrics 1997 100 1035 1039 9411381 10.1542/peds.100.6.1035

[B2] American Academy of Pediatrics (AAP) Work Group on Breastfeeding Breastfeeding and the use of human milk Pediatrics 2005 115 496 50615687461

[B3] James DCS Lessen R Position of the American Dietetic Association: promoting and supporting breastfeeding J Am Diet Assoc 2009 109 1926 1942 19862847 10.1016/j.jada.2009.09.018

[B4] US Department of Heath and Human Services Healthy People 2010 2000

[B5] Breastfeeding Report Card--United States, 2009 http://www.cdc.gov/breastfeeding/data/report_card.htm

[B6] US Department of Health and Human Services Healthy People 2020 2010

[B7] IBLCE in the Americas: Facts and Figures http://americas.iblce.org/facts-and-figures

[B8] IBCLC Care Award Directory http://www.ibclccare.org/directory.html

[B9] Centers for Disease Control and Prevention (CDC) Racial and Ethnic Differences in Breastfeeding Initiation and Duration, by State - National Immunization Survey, United States, 2004--2008 Morbidity and Mortality Weekly Report (MMWR) 2010 59 11 20339344

[B10] Becker J Kovach AC Gronseth DL Individual empowerment: how community health workers operationalize self-determination, self-sufficiency, and decision-making abilities of low-income mothers J Community Psychol 2004 32 327 342 10.1002/jcop.20000

[B11] Dennis CL Peer support within a health care context: a concept analysis Int J Nurs Stud 2003 40 321 332 10.1016/S0020-7489(02)00092-5 12605954

[B12] Garcia YE Metha A Perfect MC McWhirter JJ A senior peer counseling program: evaluation of training and benefits to counselors Educ Gerontol 1997 23 329 344 10.1080/0360127970230403

[B13] Baksi AK Al-Mrayat M Hogan D Whittingstall E Wilson P Wex J Peer advisers compared with specialist health professionals in delivering a training programme on self-management to people with diabetes: a randomized control trial Diabet Med 2008 25 1076 1082 10.1111/j.1464-5491.2008.02542.x 18937675 PMC2613236

[B14] Hilfinger Messias D Moneyham L Vyavaharkar M Murdaugh C Phillips K Embodied work: insider perspectives on the work of HIV/AIDS peer counselors Health Care Women Int 2009 30 570 592 10.1080/07399330902928766 PMC2729058 19492204

[B15] Taylor T Serrano E Anderson J Management issues related to effectively implementing a nutrition education program using peer educators J Nutr Educ 2001 33 284 292 10.1016/S1499-4046(06)60293-5 12031179

[B16] Khan N Nasti C Evans E Peer education, Exercising, and Eating Right (PEER): training of peers in an undergraduate faculty teaching partnership J Nutr Educ Behav 2009 41 68 70 10.1016/j.jneb.2008.03.116 19161924

[B17] Mastroleo N Mallett K Turrisi R Ray A Psychometric properties of the Peer Proficiency Assessment (PEPA): a tool for evaluation of undergraduate peer counselors' motivational interviewing fidelity Addict Behav 2009 34 717 722 10.1016/j.addbeh.2009.04.008 19435653 PMC2697274

[B18] Giese-Davis J Bliss-Isberg C Carson K Star P Donaghy J Cordova MJ Stevens N Wittenberg L Batten C Spiegel D The effect of peer counseling on quality of life following diagnosis of breast cancer: an observational study Psychooncology 2006 15 1014 1022 10.1002/pon.1037 16555366

[B19] Gross SR AK Cross-Barnet C Nanda JP Augustyn M Paige DM The differential impact of WIC peer counseling programs on breastfeeding initiation across the state of Maryland J Hum Lact 2009 25 435 443 10.1177/0890334409342070 19652195

[B20] Mitra A Khoury A Carothers C Foretich C Evaluation of a comprehensive Loving Support Program among state Women, Infants, and Children (WIC) program breast-feeding coordinators South Med J 2003 96 168 171 10.1097/01.SMJ.0000053675.41623.15 12630643

[B21] Anderson AK Damio G Young S Chapman DJ Pérez-Escamilla R A randomized trial assessing the efficacy of peer counseling on exclusive breastfeeding in a predominantly Latina low-income community Arch Pediatr Adolesc Med 2005 159 836 841 10.1001/archpedi.159.9.836 16143742

[B22] Chapman DJ Morel K Anderson AK Damio G Perez-Escamilla R Breastfeeding peer counseling: from efficacy through scale-up J Hum Lact 2010 26 314 326 10.1177/0890334410369481 20715336 PMC3115698

[B23] Bolton TC T Benton PA Olson BH Characteristics associated with longer breastfeeding duration: an analysis of a peer counseling support program J Hum Lact 2009 25 18 27 10.1177/0890334408325985 18971503

[B24] Chung M Raman G Trikalinos T Lau J Ip S Interventions in primary care to promote breastfeeding: an evidence review for the U.S. Preventive Services Task Force Ann Intern Med 2008 149 565 582 18936504 10.7326/0003-4819-149-8-200810210-00009

[B25] Schafer E Vogel M Viegas S Hausafus C Volunteer peer counselors increase breastfeeding duration among rural low-income women BIRTH 1998 25 101 106 10.1046/j.1523-536x.1998.00101.x 9668744

[B26] Wong E Nelson E Choi K Wong K Ip C Ho L Evaluation of a peer counselling programme to sustain breastfeeding practice in Hong Kong Int Breastfeed J 2007 2 1 11 10.1186/1746-4358-2-1 17883851 PMC2064904

[B27] Rossman B Breastfeeding peer counselors in the United States: helping to build a culture and tradition of breastfeeding J Midwifery Womens Health 2007 52 631 637 10.1016/j.jmwh.2007.05.006 17984001

[B28] Agrasada G Gustafsson J Kylberg E Ewald U Postnatal peer counselling on exclusive breastfeeding of low-birthweight infants: A randomized, controlled trial Acta Paediatr 2005 94 1109 1115 10.1080/08035250510025752 16188857

[B29] Morrow AL Community-based strategies for breastfeeding promotion and support in developing countries 2003 World Health Organization

[B30] WIC Breastfeeding Peer Counseling Training Manual http://www.cdph.ca.gov/programs/wicworks/Pages/WICBFPeerCounselor.aspx

[B31] La Leche League International Breastfeeding Peer Counselor Program Curriculum 2005 957 N Plum Grove Road, Schaumburg, IL 60173

[B32] Bronner Y Barber T Vogelhut J Resnik A Breastfeeding peer counseling: results from the national WIC survey J Hum Lact 2001 17 119 125 10.1177/089033440101700205 11847825

[B33] Raisler J Against the odds: breastfeeding experiences of low income mothers J Midwifery Womens Health 2000 45 253 263 10.1016/S1526-9523(00)00019-2 10907335

[B34] Walker SA Avis M Common reasons why peer education fails J Adolesc 1999 22 573 577 10.1006/jado.1999.0250 10469520

[B35] Definition paraprofessional http://dictionary.reference.com/browse/paraprofessional

[B36] Kistin N Abramson R Dublin P Effect of peer counselors on breastfeeding initiation, exclusivity, and duration among low-income urban women J Hum Lact 1994 10 11 15 10.1177/089033449401000121 7619241

[B37] Olson B Haider S Vangjel L Bolton T Gold J A quasi-experimental evaluation of a breastfeeding support program for low income women in Michigan Matern Child Health J 2010 14 86 93 10.1007/s10995-008-0430-5 19082697

[B38] Turner-Maffei C Lactation resources for clinicians J Midwifery Womens Health 2007 52 e57 e65 10.1016/j.jmwh.2007.03.024 17983982

[B39] Morrow AL Community-based strategies for breastfeeding promotion and support in developing countries Community-based strategies for breastfeeding promotion and support in developing countries 2003 (World Health Organization). Geneva, Switzerland

[B40] Haider R Ashworth A Kabir I Huttly SRA Effect of community-based peer counsellors on exclusive breastfeeding practices in Dhaka, Bangladesh: a randomised controlled trial Lancet 2000 356 1643 1647 10.1016/S0140-6736(00)03159-7 11089824

[B41] Davies-Adetugbo AA Adetugbo K Orewole Y Fabiyi AK Breast-feeding promotion in a diarrhoea programme in rural communities J Diarrhoeal Dis Res 1997 15 161 166 9473880

[B42] Chapman DJ Damio G Young S Perez-Escamilla R Effectiveness of breastfeeding peer counseling in a low-income, predominantly Latina population: a randomized controlled trial Arch Pediatr Adolesc Med 2004 158 897 902 10.1001/archpedi.158.9.897 15351756

[B43] Morrow AL Guerrero ML Shults J Calva JJ Lutter C Bravo J Ruiz-Palacios G Morrow RC Butterfoss FD Efficacy of home-based peer counselling to promote exclusive breastfeeding: a randomised controlled trial Lancet 1999 353 1226 1231 10.1016/S0140-6736(98)08037-4 10217083

[B44] Breastfeeding Report Card--United States 2010 http://www.cdc.gov/breastfeeding/pdf/BreastfeedingReportCard2010.pdf

[B45] Breastfeeding Among U.S. Children Born 1999-2007, CDC National Immunization Survey 2007 http://www.cdc.gov/breastfeeding/data/NIS_data/index.htm

